# Room-Temperature NH_3_ Gas Surface Acoustic Wave (SAW) Sensors Based on Graphene/PPy Composite Films Decorated by Au Nanoparticles with ppb Detection Ability

**DOI:** 10.3390/polym15224353

**Published:** 2023-11-08

**Authors:** Chi-Yen Shen, Tien-Tsan Hung, Yao-Wei Chuang, Shao-Kai Lai, Chi-Ming Tai

**Affiliations:** 1 Department of Electrical Engineering, I-Shou University, Kaohsiung 84001, Taiwan; cyshen@isu.edu.tw (C.-Y.S.); aass987642@yahoo.com.tw (Y.-W.C.); kevinlay13@gmail.com (S.-K.L.); 2 Department of Chemical Engineering, I-Shou University, Kaohsiung 84001, Taiwan; 3 Division of Gastroenterology and Hepatology, Department of Internal Medicine, E-Da Hospital, I-Shou University, Kaohsiung 82445, Taiwan; ed102166@edah.org.tw; 4 School of Medicine for International Students, College of Medicine, I-Shou University, Kaohsiung 82445, Taiwan

**Keywords:** surface acoustic wave, ammonia, AuNPs–G/PPy, sensitivity, selectivity

## Abstract

Exhaled human breath analysis has great potential for the diagnosis of diseases in non-invasive way. The ^13^C-Urea breath test for the diagnosis of Helicobacter pylori infection indicates the ammonia concentration of 50–400 ppb in the breath. This work successfully developed a surface acoustic wave (SAW) resonator based on graphene/polypyrrole composite films decorated by gold nanoparticles (AuNPs–G/PPy) with sensitivity and selectivity to detect ammonia in parts-per-billion concentrations, which is promising for the accurate diagnosis of *H. pylori* infection. XRD, EDS, and SEM characterized the AuNPs–G/PPy nanocomposites, providing comprehensive insights into their structural, compositional, and morphological properties. The gas-sensing capabilities of the fabricated SAW sensors were extensively investigated, focusing on their response to NH_3_ gas at ambient temperature. The concentration of ammonia gas was effectively quantified by monitoring the frequency shift of the SAW device. Notably, our developed SAW sensor demonstrated outstanding sensitivity, selectivity, repeatability, and reproducibility for 50–1000 ppb NH_3_ in dry air. The excellent sensing performance of the AuNPs–G/PPy hybrid composite film can be attributed to the synergistic effects of graphene’s superior conductivity, the catalytic properties of gold nanoparticles, and the conductivity sensitization facilitated by electron-hole recombination on the polypyrrole surface.

## 1. Introduction

Ammonia is a toxic and corrosive gas, capable of causing varying degrees of harm to humans and industrial settings. Ammonia corrodes pipelines within factories, thereby compromising both workplace safety and product quality. The Occupational Safety and Health Administration (OSHA) in the United States has set the permissible exposure limit for ammonia at 25 ppm for an 8 h period and 35 ppm for a 15 min duration. Inhalation of gas exceeding 500 ppm for 30 continuous minutes adversely affects the central nervous system [1]. The maximum allowable concentration of ammonia gas in both occupational and living environments, as defined by OSHA, is 35 ppm [2]. Prolonged exposure to concentrations of ammonia gas exceeding 50 ppm can result in respiratory and ocular damage, while exposure to concentrations exceeding 5000 ppm can cause sudden fainting or death. Consequently, the presence of gas sensors capable of monitoring low concentrations of ammonia [3] is paramount for ensuring a safe working and living environment, as well as saving lives.

Among various available sensing techniques, such as potential, current, chemical resistance, calorimetry, and optics, the utilization of surface acoustic waves (SAWs) for gas detection presents distinct advantages due to its rapid response, recovery speed, and the ability to perform wireless sensing in hard-to-reach areas. The origin of SAW technology traces back to 1885, when Lord Rayleigh analyzed the propagation patterns of elastic waves in isotropic materials, which are referred to as Rayleigh waves. However, the primary application of Rayleigh waves was limited to seismic studies until the 1960s due to the lack of suitable and efficient transducers. In 1965, the excitation and reception of SAWs using metal electrodes became feasible after R.M. White and F.W. Voltmer published their findings on the excitation of oscillating electric fields through paired metal electrodes. This led to the creation of interdigital transducers (IDT) and the effective excitation of SAWs by fabricating IDTs on piezoelectric substrates, marking the advancement of SAW research. In the past decade, with the advancements in micro-electromechanical systems (MEMS) technology, SAWs have found widespread application due to their high sensitivity to analytes’ physical and chemical changes. The fundamental principle of SAW sensors lies in analyte adsorption on the sensing film, which causes changes to the surface acoustic wave propagation characteristics. With the increasing breadth of sensing applications, SAW sensor design expanded to detect both gases and liquids, such as acoustic plate-mode (APM), Love wave, and surface transverse wave (STW) devices [4,5,6,7,8].

The commonly used sensing materials exhibiting good response to ammonia gas include conductive polymers [9,10], metal oxide semiconductors [11], and composite materials [12,13]. Sensors utilizing semiconductor metal oxide materials typically require higher operating temperatures to exhibit a significant response to the target gas, and they have poorer recovery abilities. In contrast, sensors using conductive polymers operate at room temperature but only exhibit a noticeable response at high analyte concentrations, with longer recovery times. Due to the limitations of individual sensing materials, developing new composite materials as sensing materials has become a trend in recent years. These include composite materials of conductive polymer/metal oxide [14,15], conductive polymer/metal oxide/graphene oxide (GO) [16,17], conductive polymer/graphene (G) [18,19], and conductive polymer/metal oxide/reduced graphene oxide (rGO) [20]. In particular, composites of graphene and traditional sensing materials such as noble metals, metal oxides, and conductive polymers not only retain individual characteristics but also exhibit additional new properties.

Previous findings have shown PPy-conducting polymer films exhibit high sensitivity to NH_3_ and excellent signal recovery [12,21]. Graphene is used as a sensing material due to its high specific surface area and unique electrical properties such as high mobility and low noise [22]. Gold nanoparticles have been utilized to modify electrochemical electrodes made from GO or rGO/metal oxide nanocomposites, further enhancing their sensitivity and selectivity towards the target gas [23,24]. Combining PPy, graphene, and gold nanoparticles effectively leverages the unique attributes of each material while mitigating their respective limitations. PPy offers selectivity, graphene provides high surface area and conductivity, and gold nanoparticles contribute to catalytic activity. The synergy of these three materials results in a sensor with improved sensitivity, selectivity, and response speed for NH_3_ detection. Their distinct properties also enable a more comprehensive analysis of the NH_3_ concentration and reduce the risk of false readings due to interfering gases. Hence, this study focuses on the preparation of AuNPs–G/PPy hybrid nanocomposite sensing films through in situ chemical oxidation polymerization of AuNPs, graphene, and pyrrole, along with the incorporation of low-loss surface acoustic wave devices. The combination of these elements results in a highly sensitive SAW sensor capable of detecting NH_3_ at ppb-level concentrations at room temperature. The sensors thus show promising potential in detecting parts per billion-level NH_3_, which may open up new applications for the accurate diagnosis of *H. pylori* infection.

## 2. Materials and Methods

### 2.1. Materials and Reagents

Graphene and AuNPs (core size: 20 nm ± 2 nm) were purchased from Acros (Bergen County, NJ, USA). The pyrrole monomer was obtained from Acros and purified through distillation at reduced pressure before use. Other reagents, including PSSA (ALFA) and ammonium peroxydisulfate (APS; Showa, Gyoda, Japan), were used without further purification. All chemicals used here were of analytical reagent grade. NH_3_ gas (50 and 1000 ppb) was obtained from Jing-De Gas Co. (Kaohsiung, Taiwan).

### 2.2. Preparation of AuNPs–G/PPy Hybrid Nanocomposite Film

**Figure 1:**
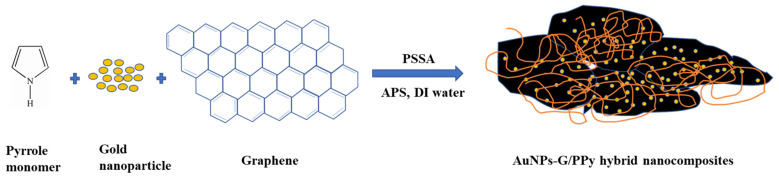
Schematic illustration for the preparation of AuNPs–G/PPy hybrid nanocomposite.

Figure 1 illustrates the process of AuNPs–G/PPy hybrid nanocomposite preparation. The AuNPs–G/PPy hybrid nanocomposite was synthesized through in situ chemical oxidative polymerization. First, 13.3 mL of PSSA and 2.0 g of AuNPs in 16.7 mL of distilled water were added to a reaction vessel containing a stirrer. Then, 0.3 g of graphene was mixed with the 0.1 g of surfactant solution (SDS) and ultrasonicated for 3 h to form a soft template in solution. Freshly distilled pyrrole monomer (0.5 g) was slowly added dropwise into the aforementioned solution, with continuous stirring for 30 min in an ice bath. Next, 2.0 g of APS in 10 mL of distilled water was slowly added into this solution. The polymerization process lasted 3 h at about 5 °C with constant mechanical stirring. The synthesized AuNPs–G/PPy hybrid nanocomposite was filtered and rinsed several times with distilled water and methanol. The obtained powder was vacuum dried at 60 °C for 24 h. The obtained powder of AuNPs–G/PPy hybrid nanocomposite was mixed with appropriate amounts of distilled water to prepare AuNPs–G/PPy hybrid nanocomposite sensitive films using spin coating. The surface morphology and composition of these nanocomposite powder and films were characterized on an environmental scanning electron microscope (ESEM, Quanta 200, FEI, Hillsboro, OR, USA) equipped with an energy dispersive X-ray spectroscope (EDS). The crystalline structure and resistivity of these nanocomposite films were characterized and measured on an X-ray diffraction (XRD, Siemens D5000, Bruker, Mannheim, Germany) and a Hall effect measurement system (HMS-3000, Ecopia, Anyang-si, Republic of Korea), respectively.

### 2.3. SAW Sensor Fabrication

**Figure 2:**
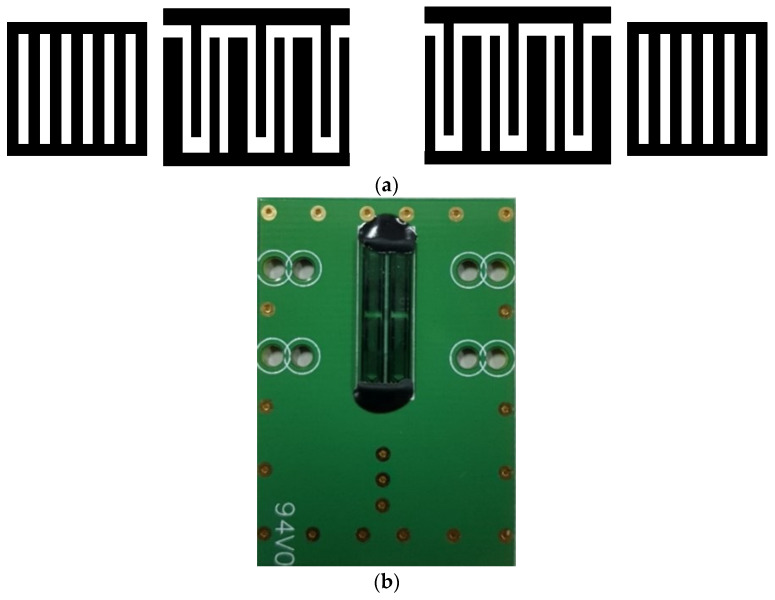
Schematic illustration of (**a**) two-port resonators consisting of EWC/SPUDT IDT and (**b**) SAW device.

Two-port SAW resonators fabricated on a ST-cut quartz substrate were used to detect NH_3_ gas. A dual-track configuration was used to reduce interference from the environment. The input/output IDTs in each channel adopted the electrode-width-controlled single-phase unidirectional transducer (EWC/SPUDT) structure and were combined with the reflection grating on both sides of the channel to form a two-port resonator. Figure 2 presents a top view of the EWC/SPUDT IDT and a dual-track configuration of SAW resonators employed in this study, which are the same as in our previous study [13]. A sensing track was produced by spin-coating a 1.5 × 0.5 mm^2^ sensitive area of the AuNPs–G/PPy hybrid nanocomposite layer in between two IDTs, such that the reference track surface was free. The thickness of the AuNPs–G/PPy hybrid nanocomposite film was measured using an optical thin-film measurement instrument (TF-166, New Span Opto-Technology Inc., Miami, FL, USA), and the thickness of the sensing film was approximately 130 nm.

### 2.4. Gas Sensing Measurements

**Figure 3:**
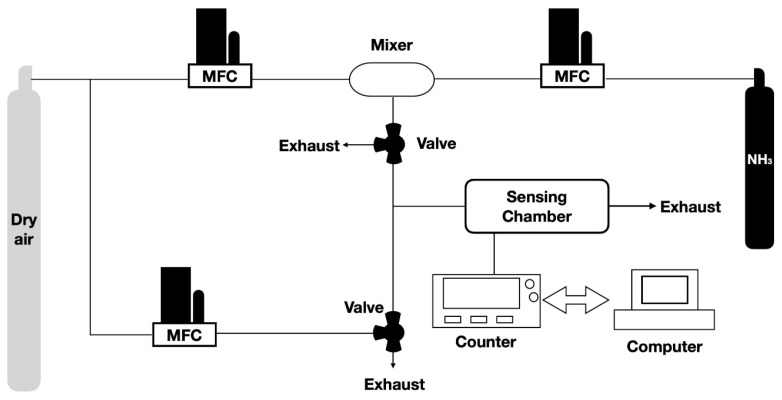
Experimental setup for ammonia gas sensing measurement.

The sensing properties of the fabricated sensors were measured within an enclosure containing various concentrations of NH_3_ gas. Mass flow controllers (MFC, Sierra, Kyoto, Japan) were used to produce the required gas dilutions using certified 2 ppm NH_3_ and dry air cylinders (Jing-De Gas, Kaohsiung, Taiwan). The NH_3_ and dry air were transported using an MFC to change the NH_3_ gas-to-dry ratio. A NH_3_ sensor (FENO, Bedfont, UK) was used to independently confirm NH_3_ gas concentration for gases generated using the aforementioned dilution method. The outflow was maintained at a constant rate of 110 mL/min during the measurements. The dual-track sensor was placed in a temperature-stabilized, sealed 5 cm^3^ sensing chamber integrated with the oscillation circuit. A temperature controller kept experiments at a constant temperature of 24 °C. Figure 3 illustrates the experimental system under dry conditions.

The frequency changes of the SAW sensor were measured using a frequency counter (53132A, Agilent, Santa Clara, CA, USA). Firstly, dry air was introduced into the sensing chamber for 30 min, to stabilize the experimental environment and electrical signals. NH_3_, as the sensing gas, was mixed with dry air (carrier gas), and the desired concentration was controlled using an MFC. The gas mixture was allowed to blend for at least 30 min to ensure homogeneous mixing of gases. The gas valve was then switched to introduce the desired concentration of NH_3_ gas into the sensing chamber, and the sensing time was set to 3 min. After 3 min, the valve was switched back, and dry air was continuously supplied to the sensing chamber for 30 min to complete one cycle (approximately 1 h). After the experiments, the sensor was stored in a sealed container filled with nitrogen gas to prevent contamination or moisture absorption by the sensing film.

## 3. Results and Discussion

### 3.1. Material Analysis of the AuNPs–G/PPy Hybrid Nanocomposite

**Figure 4:**
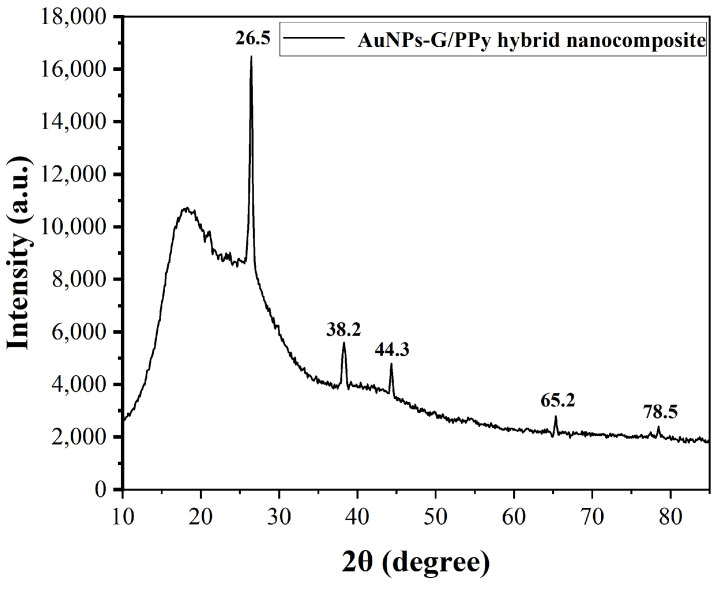
XRD pattern of the AuNPs–G/PPy hybrid nanocomposite on glass substrate.

Figure 4 shows the XRD patterns of the AuNPs–G/PPy hybrid nanocomposite. As can be seen, one broad peak at 2*θ* = 10°~30° may be ascribed to the doped PPy chains [25]. The broad peak is due to the scattering of the PPy chains at the interplanar spacing. The graphene samples showed a main reflection peak at 26.5°, which could be indexed to the characteristic peak reflections of graphite from the graphene (JCPDS No. 01-0646) [26]. The additional four sharp diffraction peaks centered at 2*θ* = 38.2°, 44.3°, 65.2°, and 78.5° were due to Bragg’s reflections from the (111), (200), (220), and (311) planes of the face-centered cubic Au, respectively (JCPDS card No. 004-0784) [27].

**Figure 5:**
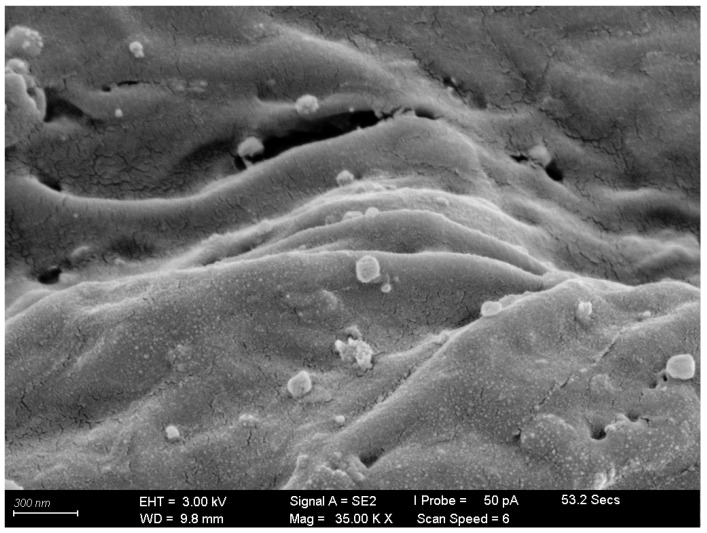
Top view SEM images of the AuNPs–G/PPy hybrid nanocomposite film (35,000×).

**Figure 6:**
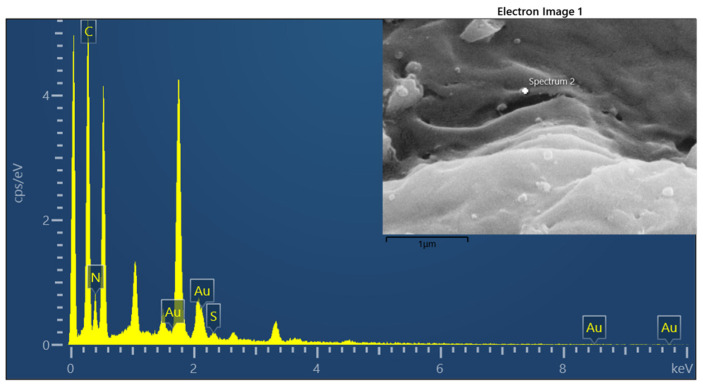
EDS mapping of the AuNPs–G/PPy hybrid nanocomposite film.

The SEM image of the AuNPs–G/PPy hybrid nanocomposite in Figure 5 shows the uniformly dispersed spherical AuNPs decorating the large graphene sheets. It can be clearly seen that well-dispersed small gold nanoparticles were in a strong interaction with graphene/PPy, and the average diameters of the gold nanoparticles were about 20 nm. All these results strongly confirm the successful preparation of AuNPs–G/PPy hybrid nanocomposites using an in situ chemical method. Figure 5 also shows various wrinkle patterns on the film surface, which increased the overall adsorption surface area, enabling effective ammonia adsorption. Figure 6 displays the EDS analysis of the AuNPs–G/PPy nanocomposite film, confirming the distribution of elements such as C, N, S, and Au, consistent with the expected material characteristics.

The Hall measurement analysis instrument was utilized to measure the resistivity of the AuNPs–G/PPy hybrid nanocomposite film. It was observed that the original resistivity of the AuNPs–G/PPy nanocomposite film was 8.706 × 10^−3^ Ω/cm. However, after the sensing film adsorbed 800 ppb NH_3_ gas, the resistivity increased to 1.329 × 10^−1^ Ω/cm. The increased resistivity of AuNPs–G/PPy nanocomposite film upon NH_3_ exposure indicates P-type semiconductor characteristics. The SEM images in Figure 5, magnified at 45,000×, reveal the wrinkled multilayer structure of PPy, which resulted from its polymerization between the large graphene sheets, facilitating charge carrier conduction between the graphene and PPy [28]. The presence of spherical AuNPs attached to the graphene surface can also be observed in Figure 5.

### 3.2. Gas Sensing Properties

The SAW sensors were coated with a sensing layer for chemical sensing. Any changes in the mass, mechanical, or electrical properties of this sensing layer upon exposure to the foreign molecules can perturb the surface acoustic waves, enabling the devices to be used as sensors [29]. The perturbation from the wave propagation characteristics after gas adsorption can be written as

(1) $$\frac{\Delta f}{f_{0}}≅\frac{\Delta v}{v_{0}}=-c_{m}f_{0}\Delta \left(\frac{m}{A}\right)+4c_{e}f_{0}\left(\Delta hG^{\prime }\right)-\frac{K^{2}}{2}\Delta \frac{1}{{\left(v_{0}C_{s}/\sigma_{s}\right)}^{2}+1}$$

where $c_{m}$ and $c_{e}$ are the coefficients of mass sensitivity and elasticity, respectively; m/A is the change in mass per unit area; h is the thickness of the sensitive layer; $G^{\prime }$ is the shear modulus; $K^{2}$ is the electromechanical coupling coefficient; and $\sigma_{s}$ is the sheet conductivity of the sensitive layer. The first term on the right-hand side of Equation (1) represents the mass-loading effect that results in negative frequency shifts and is a function of the NH_3_ gas concentration. The second and the third term are the contribution of the elastic properties and the acoustoelectric effect of the sensitive layer, respectively, and produce a positive frequency shift because NH_3_ is a reducing gas [30].

**Figure 7:**
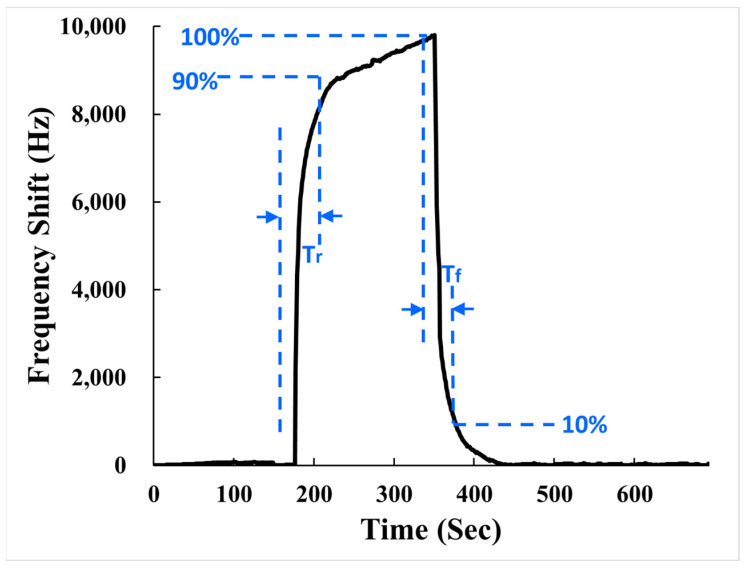
Frequency transient response of the SAW sensor with AuNPs–G/PPy sensing film to 1 ppm NH_3_ in dry air at room temperature.

The sensing performance of the SAW sensors was evaluated in terms of analytical validations such as sensitivity, limit of detection (LOD), repeatability, stability, and selectivity. These analytical parameters are essential to demonstrate the quality and reliability of the sensor. Figure 7 shows the frequency transient response of the SAW sensor with AuNPs–G/PPy sensing film to 1 ppm NH_3_ in a dry air environment. Since NH_3_ gas is a reducing gas, the acoustoelectric effect and elastic effect exhibit a positive frequency change, while the mass loading induces a negative frequency change. Figure 7 shows that the SAW sensor with AuNPs–G/PPy sensing film generated a positive frequency change when detecting NH_3_, indicating that the sum of the acoustoelectric effect and elastic effect was greater than the mass loading. It indicates that the AuNPs–G/PPy hybrid nanocomposite film exhibited an increase in electrical resistance when detecting NH_3_ gas. This led to a positive response in the third term on the right side of Equation (1), which, when combined with the second term of the elastic effect, was greater than the negative change caused by the first term of the mass loading. Consequently, the SAW sensor demonstrated a positive frequency response, as Figure 7 confirms.

**Figure 8:**
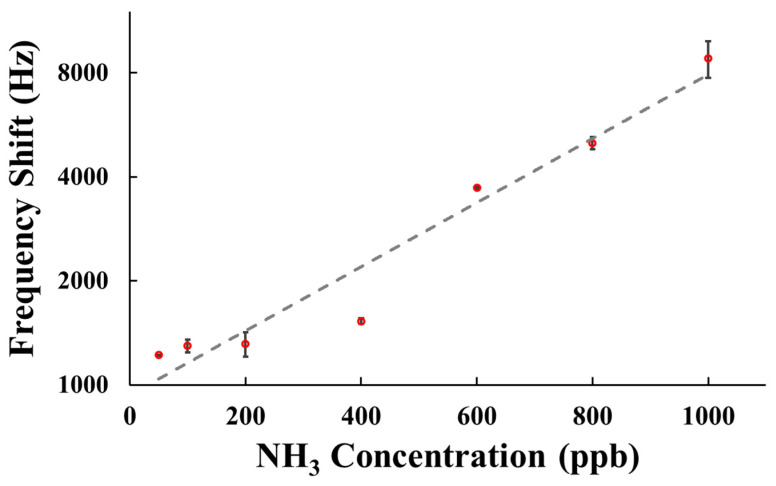
Frequency shifts of a SAW sensor coated with AuNPs–G/PPy sensing film to various concentrations of NH_3_ gas in dry air at room temperature.

Figure 8 illustrates the frequency shift of the SAW sensor coated with AuNPs–G/PPy sensing film when detecting NH_3_ concentrations ranging from 50 to 1000 ppb in a dry air environment. The data presented for each concentration represents the results of three experimental measurements. As the SAW sensor detected NH_3_, the frequency shift increased with the increasing NH_3_ concentration. Figure 8 shows a linear relationship between the response of the proposed SAW sensor and NH_3_ concentration in the concentration range of 50 to 1000 ppb. The sensitivity of the SAW sensor in detecting 50 to 1000 ppb NH_3_ was 8 Hz/ppb, demonstrating excellent sensitivity for NH_3_ detection. The concentration of ammonia in the exhaled breath of healthy individuals ranges from approximately 425 ppb to 1800 ppb [31], whereas individuals with Helicobacter pylori infection typically exhibit ammonia concentrations ranging from 50 ppb to 400 ppb after undergoing the carbon-13 urea breath test [32]. Since this study aims to develop a sensor for detecting Helicobacter pylori infection, experiments involving NH_3_ concentrations higher than 1000 ppb were not conducted.

The minimum detectable limit (limit of detection, LOD) of a sensor is typically defined as the signal-to-noise ratio (S/N) of 3. In the case of the SAW sensor developed in this study, the frequency change for detecting 50 ppb NH_3_ was 1222 Hz, with noise of 17 Hz, resulting in an S/N ratio of 72. Therefore, the estimated LOD of this sensor is 3 ppb.

**Figure 9:**
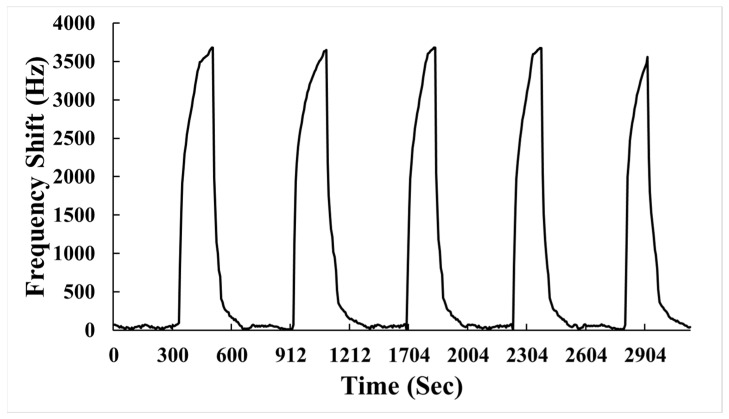
Repeatability of a SAW sensor coated with AuNPs–G/PPy sensing film exposed to 600 ppb NH_3_ gas.

A sensor with good repeatability demonstrates high reliability during the sensing process and yields consistent results under repeated operations. Figure 9 illustrates the repeatability experiment results of the AuNPs–G/PPy-coated SAW sensor for detecting 600 ppb NH_3_ in dry air. The calculation formula for repeatability is as follows:

(2) $$\mathrm{Repeatability}=\frac{{∆f}_{5}}{∆f_{1}}\times 100\%$$

where Δ*f*_1_ represents the frequency response obtained in the first experiment, and Δ*f*_5_ represents the frequency response obtained in the second experiment. Based on the Equation (2) calculation, for the AuNPs–G/PPy-coated SAW sensor in the repeatability experiment with 600 ppb NH_3_, the frequency shifts for the first and fifth experiments were 3679 Hz and 3559 Hz, respectively. This yields a repeatability of 97%, demonstrating the excellent repeatability of the AuNPs–G/PPy-coated SAW sensor. In this work, the reproducibility of the identical sensor was also tested by having two independent investigators measure the sensor response to 600 ppb NH_3_ in dry air. The frequency shifts measured were 3679 Hz and 3700 Hz for each investigator. The close alignment of results between investigators further confirms the high reproducibility of the proposed sensor.

Table 1 shows the frequency shift, response time, and recovery time of the AuNPs–G/PPy-coated SAW sensor for different concentrations of NH_3_ gas in a dry air environment. The response time (T_r_) is defined as the time required for the response frequency to increase to 90% of the maximum response after introducing NH_3_ gas (as illustrated in Figure 7). The recovery time (T_f_) is defined as the time required for the response frequency to return to 90% of the baseline after removing NH_3_ gas. It is clearly seen from Table 1 that the SAW sensor coated with AuNPs–G/PPy exhibited response and recovery times within 2.5 min in a dry air environment.

Table 2 presents the long-term response characteristics of the SAW sensor coated with AuNPs–G/PPy to 50 ppb NH_3_ in a dry air environment at room temperature. On day 1, the frequency change was 1273 Hz. By day 20, it had decreased to 1219 Hz, and by day 30, it had further reduced to 563 Hz. This indicates that the frequency change of the SAW sensor decays over time during NH_3_ detection. The long-term stability can be calculated using the following formula:

(3) $$\text{Long-term}\;\mathrm{stability}\;(\%)=\frac{{∆f}_{n}}{{∆f}_{1}}\times 100\%$$

where Δ*f_n_* represents the frequency shift on the nth day. For this calculation, Δ*f*_1_ is the frequency shift on the first day (1273 Hz), and Δ*f*_30_ is the frequency shift on the 30th day (563 Hz). Using the formula, the 20-day long-term stability of the AuNPs–G/PPy-coated SAW sensor is calculated as 96%, while the 30-day stability is 44%. The sensor exhibited stable long-term response within the first 20 days but experienced a rapid decline after 20 days.

**Figure 10:**
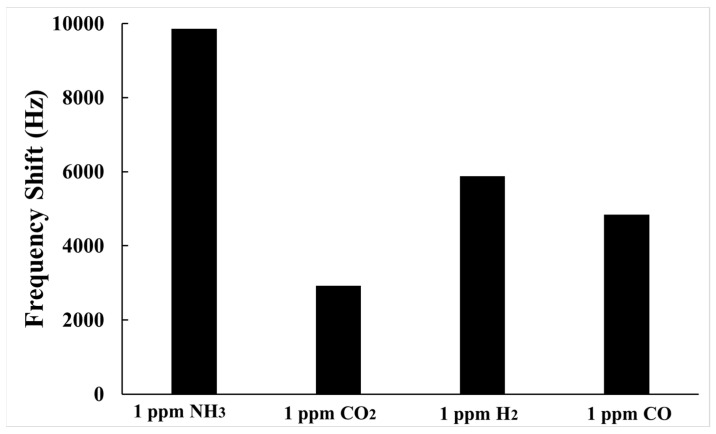
Frequency shifts of a SAW sensor coated with AuNPs–G/PPy sensing film towards 1.0 ppm NH_3_ gas, 1.0 ppm CO_2_ gas, 1.0 ppm H_2_ gas, and 1.0 ppm CO gas.

Selective analysis of gas sensors is crucial to demonstrate whether the SAW sensor responds to specific gases without being affected by other gases. In this study, 1 ppm H_2_, 1 ppm CO, and 1 ppm CO_2_ were tested as interfering gases. Figure 10 illustrates the frequency shift for various gases. The selectivity can be calculated using the following formula:

(4) $$\mathrm{Selectivity}=\frac{{∆f}_{{NH}_{3}}}{{∆f}_{int}}\times 100\%$$

where Δ*f*_*NH*_3__ and Δ*f_int_* represent the frequency shift of the sensor to NH_3_ and the interfering gases, respectively. It is clearly seen from Figure 10 that the frequency shift for 1 ppm NH_3_ gas was over 1.5 times higher than for 1 ppm H_2_, 1 ppm CO, and 1 ppm CO_2_. Accordingly, the SAW sensor coated with AuNPs–G/PPy hybrid nanocomposite film can selectively detect ammonia at ppm levels in dry air at room temperature despite the presence of common interfering gases.

Table 3 shows some of the NH_3_ sensors operating at room temperature that are reported in the literature [11,14,15,18,21,33,34,35]. Compared to other ammonia sensors, the apparent frequency response in the present work is rapidly towards ppb-level NH_3_. Hence, the present EWC/SPUDT SAW sensor coated with AuNPs–G/PPy hybrid nanocomposite film is able to sensitively detect NH_3_ concentrations of the order of parts per-billion at room temperature.

### 3.3. Mechanism of Gas Sensing

**Figure 11:**
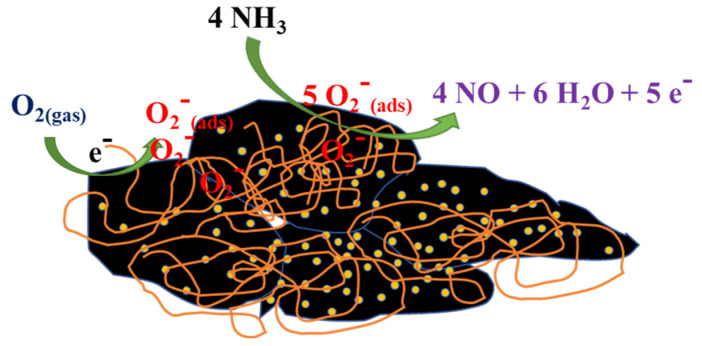
The sensing mechanism illustration of the AuNPs–G/PPy hybrid nanocomposite film.

The P-type semiconductor nature of the AuNPs–G/PPy hybrid nanocomposite film, enhanced by graphene’s high electron mobility at room temperature, facilitates rapid carrier transport within the film, resulting in improved sensing characteristics. Furthermore, graphene’s large surface area and wrinkled multilayer structure provide favorable conditions for ammonia molecule adsorption on the sensing film surface [36]. In dry air, oxygen molecules spontaneously adsorb on the gold nanoparticles, graphene, and PPy surface, capturing electrons to form reactive electrophilic oxygen ions, O_2_^−^. Upon exposure to NH_3_, two simultaneous processes occur in the AuNPs–G/PPy hybrid nanocomposite film. On one hand, NH_3_ molecules react with O_2_^−^ to generate a large number of electrons, as shown in the following equation: 4 NH_3_(g) + 5 O_2_^−^_(ads)_ → 4 NO(g) + 6 H_2_O(g) + 5e^−^ [37]. Figure 11 illustrates the schematic representation of the sensing mechanism.

On the other hand, an oxidation–reduction reaction takes place between PPy and NH_3_, as illustrated by the following equations:

Adsorption: PPy^+^ + NH_3_ → PPy^0^ + NH_4_^+^

Desorption: PPy^0^ + NH_4_^+^ → PPy^+^ + NH_3_

When NH_3_ molecules encounter PPy, ammonia molecules lose electrons, transferring them to PPy forming ammonium ions, leading to an increase in PPy’s resistivity. Upon the removal of NH_3_ gas and renewal with air, the conductivity of the AuNPs–G/PPy nanocomposite film can be restored. Additionally, as suggested in the literature [38], there may be π–π stacking between the graphene and PPy layers, allowing for electron transfer between them. The electrons generated from the reaction between NH_3_ and O_2_^−^ enter the PPy layer, further promoting the redox reaction between PPy and NH_3_. This results in the deprotonation of P-type PPy and a reduction in charge carriers in the PPy main chain. Finally, the electrons rapidly transfer to the AuNPs–G/PPy nanocomposite film and recombine with the holes in the P-type semiconductor. These simultaneous processes of electron and hole recombination in the PPy and graphene contribute to a decrease in hole density and an increase in resistance, particularly pronounced at higher concentrations of NH_3_. Compared to the response process, the AuNPs–G/PPy nanocomposite film quickly loses electrons by reacting with adsorbed oxygen molecules, thereby returning to the baseline position rapidly. The presence of AuNPs allows for more NH_3_ gas molecules to adsorb onto the surface of the graphene, as NH_3_ gas molecules can strongly bind to the surface. This indirectly enhances the adsorption capacity of graphene for NH_3_ gas molecules, consequently improving the sensitivity of NH_3_ detection [39].

### 3.4. Humidity Effect

**Figure 12:**
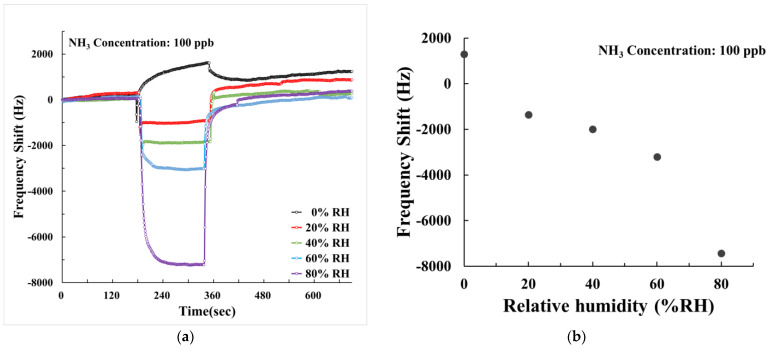
(**a**) Frequency transient responses and (**b**) frequency shifts of the AuNPs–G/PPy SAW sensor at different humidity towards 100 ppb NH_3_.

**Figure 13:**
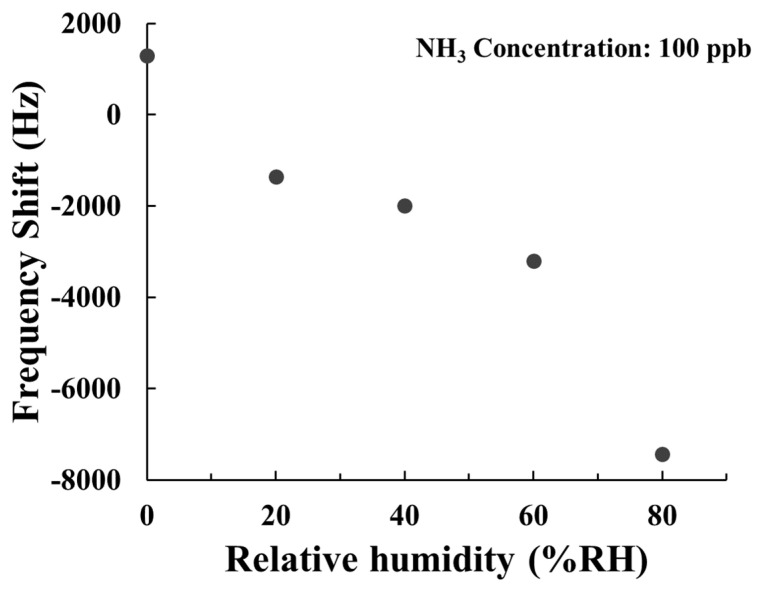
The frequency shift of the AuNPs–G/PPy SAW sensor in various humidity environments.

Considering that environmental humidity fluctuations can be significant in practical sensor applications, we investigated the influence of humidity (0~80% RH) on the sensing characteristics of our SAW sensor. Figure 12 illustrates the dynamic response of the SAW sensor with AuNPs–G/PPy composite film when exposed to 100 ppb NH_3_ at various relative humidities (RH). At 0% RH, the sensor exhibited a positive frequency response. However, in humid environments (>20% RH), the frequency response became negative due to the NH_3_ molecules’ pronounced affinity for H_2_O molecules. This negative shift increased with higher humidity levels. Within the range of 20–80% RH, the sensor demonstrated a more pronounced negative frequency change, indicating that ambient H_2_O assisted the AuNPs–G/PPy nanocomposite film in capturing more NH_3_ molecules, thereby increasing the mass loading. Furthermore, we conducted tests on the SAW sensor with AuNPs–G/PPy composite film to assess the frequency shift at different humidity levels, as depicted in Figure 13. The negative frequency response increased with rising humidity, exhibiting a similar phenomenon to that in Figure 12, albeit with a lesser response magnitude at the same humidity level. Figure 12 and Figure 13 collectively illustrate that the SAW sensor with AuNPs–G/PPy composite film exhibits a negative frequency response in humid conditions, which intensifies with rising humidity. This implies the negative mass-loading change outweighs the positive elastic and acoustoelectric effects in humid environments. Moreover, at humidity increases, the AuNPs–G/PPy nanocomposite film captures a greater quantity of NH_3_ molecules, resulting in a more substantial mass loading, a phenomenon validated by the results in Figure 12.

## 4. Conclusions

This study presents a SAW sensor based on AuNPs–G/PPy hybrid nanocomposite film for highly sensitive and rapid detection of ammonia gas at ppb levels at room temperature. The AuNPs–G/PPy sensing film was characterized by XRD, EDS, and SEM techniques. The film exhibited a wrinkled and multilayered structure, which increased the gas adsorption surface area. The interaction between graphene and PPy promoted electron conduction. Experimental results showed that the SAW sensor coated with AuNPs–G/PPy exhibited a positive frequency shift when detecting 50–1000 ppb NH_3_ in dry air, indicating that the combined elastic and acoustoelectric effects exceeded mass loading. The frequency response increased stably and linearly with rising ammonia concentration. The sensitivity of the SAW sensor in detecting 50–1000 ppb NH_3_ was 8 Hz/ppb, demonstrating the excellent NH_3_ detection. Furthermore, the SAW sensor coated with AuNPs–G/PPy showed fast response, reproducibility, and selectivity, remaining stable for 20 days. Based on these findings, the SAW sensor coated with AuNPs–G/PPy hybrid nanocomposite film developed in this study effectively detected 50–1000 ppb NH_3_ in dry air, exhibiting potential for medical diagnosis through human breath analysis. While fluctuations in environmental humidity do influence the outcomes of sensor detection, a practical approach is to dehydrate the exhaled human breath gases before undertaking subsequent ammonia concentration measurements.

## Figures and Tables

**Table 1:** Sensing response of the SAW sensor coated with AuNPs–G/PPy sensing film toward various concentrations of NH_3_ gas in dry air.

NH_3_ Concentration (ppb)	50	100	200	400	600	800	1000
Frequency shift (Hz)	1222	1353	1208	1559	3692	4808	9867
Response time (s)	128	125	120	118	98	76	61
Recovery time (s)	140	120	122	120	87	77	56

**Table 2:** Stability of a SAW sensor coated with AuNPs–G/PPy sensing film to 50 ppb NH_3_ gas for 30 days.

Time (Day)	1	10	20	30
Frequency shift (Hz)	1273	1222	1219	563

**Table 3:** Comparison of different NH_3_ sensors operating at room temperature reported in the literature.

Sensing Film	Sensitivity	Detection Limit (LOD)	Response Time	Recovery Time	Reference
SnO_2_/Co_3_O_4_	3.33 Hz/ppm	9 ppm	100–120 s	30–50 s	[11]
PANI/WO_3_	121% to 100 ppm	1 ppm	32 s	388 s	[14]
PANI/SnO_2_	29 to 100 ppm	≥1.8 ppm	31 s	-	[15]
PANI/HNTs	257.14% (50 ppm)	10 ppb	158 s	162 s	[33]
PANI–rGO	13% (15 ppm)	0.3 ppm	96 s	22.1 min	[34]
Yttrium Stabilized Zirconium (YSZ)	3.53 × 10^−14^ F/μM	2.5 ppb	-	-	[35]
PPy/G	1.7% to 1 ppm	1 ppm	2 min	5 min	[18]
PPy	12% to 20 ppm	5 ppm	20 s	20 min	[21]
AuNPs–G/PPy	8 Hz/ppb	3 ppb	128 s	140 s	This work

## Data Availability

The data presented in this study are available on request from the corresponding author.
